# A Feasibility Study for the Production of Three-dimensional-printed Spine Models Using Simultaneously Extruded Thermoplastic Polymers

**DOI:** 10.7759/cureus.4440

**Published:** 2019-04-12

**Authors:** William Clifton, Eric Nottmeier, Aaron Damon, Conrad Dove, Selby G Chen, Mark Pichelmann

**Affiliations:** 1 Neurosurgery, Mayo Clinic, Jacksonville, USA; 2 Neurosurgery, Mayo Clinic, Rochester, USA

**Keywords:** 3d-printed spine model, polymer, simulation, spine neurosurgery, spine biomechanics, pla, pva, cancellous bone

## Abstract

Background

Medical simulation is an emerging field for resident training. Three-dimensional printing has accelerated the development of models for spine surgical simulation. Previous models have utilized augmented infill ratios to simulate the density difference between cortical and cancellous bone; however, this does not fully account for differences in the material properties of these components of human vertebrae. In order to replicate the differences in both density and material characteristics for realistic spinal simulation, we created a three-dimensional model composed of multiple thermoplastic polymers.

Materials and methods

Three lumbar vertebrae and 20 C2 vertebrae models using an experimental dual material fabrication method were printed on an Ultimaker S5 3D printer. Assessment of model integrity during instrumentation as well as user tactile feedback were points of interest to determine prototype viability for educational and biomechanical use. The experimental cohort was compared with a control cohort consisting of a single material print, resin print, and polyurethane mold.

Results

Based on tactile feedback, the experimental dual material print (polylactic acid [PLA]/polyvinyl alcohol [PVA]) more accurately represented the sensation of in vivo instrumentation during pedicle probing, pedicle tapping, and screw placement. There were no instrumentation or material failures in the PLA/PVA experimental model cohort.

Conclusions

This feasibility study indicates that multiple material printing using PLA and PVA is a viable method to replicate the cortico-cancellous interface in vertebral models. This concept and design using our unique infill algorithm have not been yet reported in the medical literature. Further educational and biomechanical testing on our design is currently underway to establish this printing method as a new standard for spinal biomimetic modeling.

## Introduction

Medical simulation is an emerging field for procedural instruction in medical and surgical training programs [1]. Three-dimensional printing has accelerated the development of models for spinal simulation and instruction of surgical technique in academic institutions [2]. The current use of materials for modeling human vertebrae for training purposes and biomechanical testing is widely variable. Cadaveric tissue is currently the gold standard of spinal biomechanical testing and surgical training. However, the use of cadaveric models is fraught with high cost of the tissue as well as housing and staffing for tissue maintenance. There are also stringent regulations on the handling of cadaveric specimens, which create difficulties for both industrial and academic centers. Due to these limitations, the use of polyurethane foam molds for spinal surgical simulation and biomechanical testing is a popular choice. The cortico-cancellous interface in these models does not always reflect the in vivo tactile feel of “hard-to-soft” transition during pedicle access. The known mechanical properties of the material reflect this as well [3]. Recently, there has been an increased effort to mimic the tactile feedback of penetrating cortical bone into cancellous bone during spinal instrumentation. Simulation of the cortico-cancellous interface is the key component in creating a model that is useful for both surgical training as well as product development and marketing of spinal implants. Fused deposition modeling (FDM) and the use of thermoplastic polymers in 3D printing are current technologies that have the ability to recreate this chief feature. Previous endeavors have utilized matrixing of the inner portions of the print to replicate density differences in the cortical and cancellous bone [4]. This is typically performed with a single material for the entirety of the print. However, this technique does not accurately reflect the differences in the material composition of human cortical and cancellous bone. This gap between biology and technology has yet to be filled.

Polylactic acid (PLA) filament is a commonly used biodegradable thermoplastic used in 3D printing [5-6]. It is also commonly used in conjunction with polyvinyl alcohol (PVA). PVA is a spongy, biodegradable, water-soluble plastic that is generally used as a support material during three-dimensional printing. Its sensitive thermal and chemical properties usually do not make it suitable for stand-alone structural complexes and thus has had limited research into its use for the creation of working models. Its applications in bioengineering and tissue scaffolding have been well established [7-10]. PLA has a higher density than PVA and different elastic properties. PVA filament demonstrates a much higher elongation at break than PVA; therefore it is less brittle and has higher ductility [5,10]. These differences make these two materials appropriate to simulate the cortico-cancellous interface of vertebral bone [11]. The density of human cortical bone is higher than cancellous bone and with different elastic properties; however, there is a large distribution of normal values in human adults depending on age, race, sex, and other demographics [12-14]. This mechanical analysis was key in selecting our polymer composite.

Utilizing the individual properties of these materials, an experimental fabrication method was developed in order to create a vertebral model with multiple material compositions. Augmented “tough” PLA was chosen as the outer shell material to simulate cortical bone, and PVA was chosen to simulate spongy cancellous bone within the PLA shell. Using these materials simultaneously, we endeavored to simulate the cortico-cancellous interface and tactile response during spinal instrumentation on our models.

## Materials and methods

**Figure 1:**
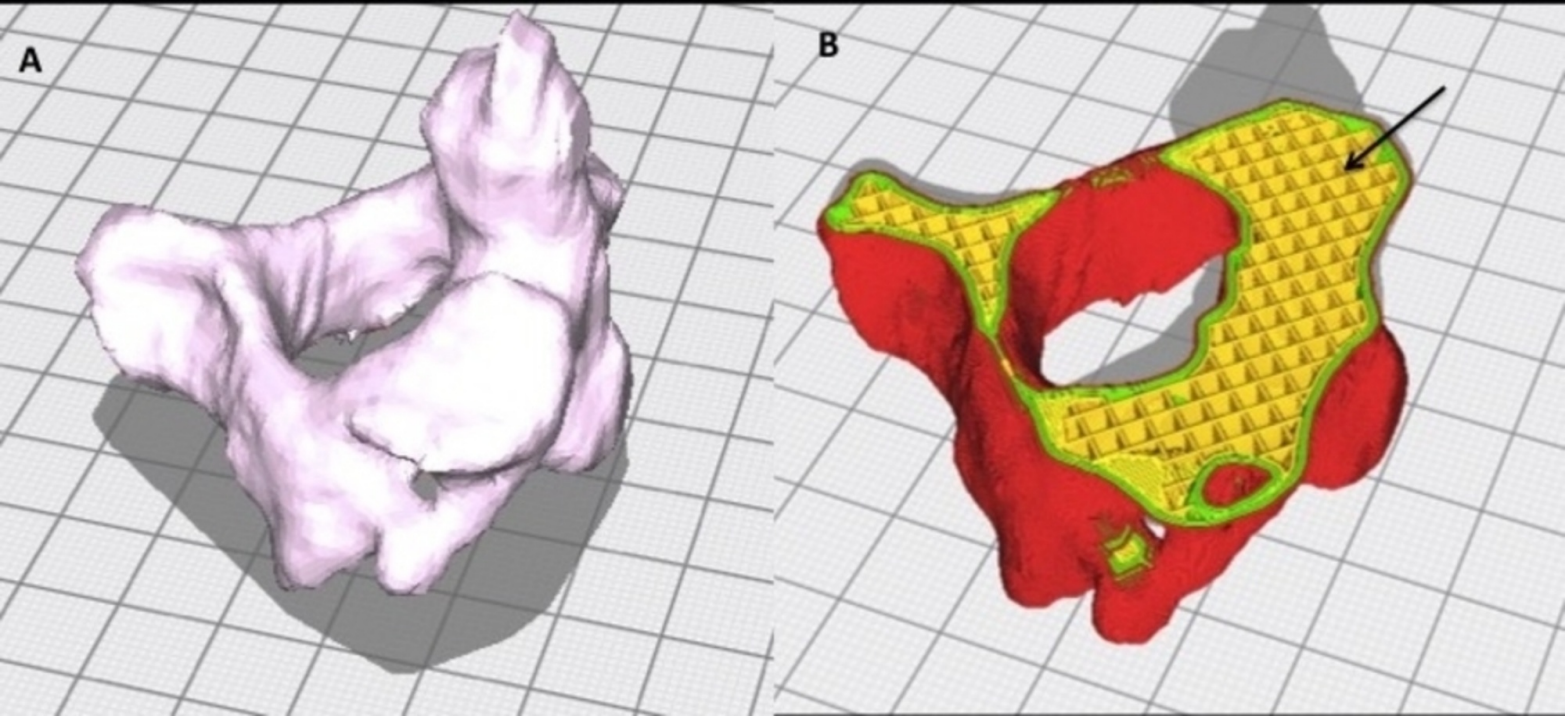
Software design of experimental C2 vertebral model (A) Solid view and (B) layered view demonstrating the planned infill matrix of the PVA (yellow, black arrow) and the outer cortical shell (red surface) PVA, polyvinyl alcohol

**Figure 2:**
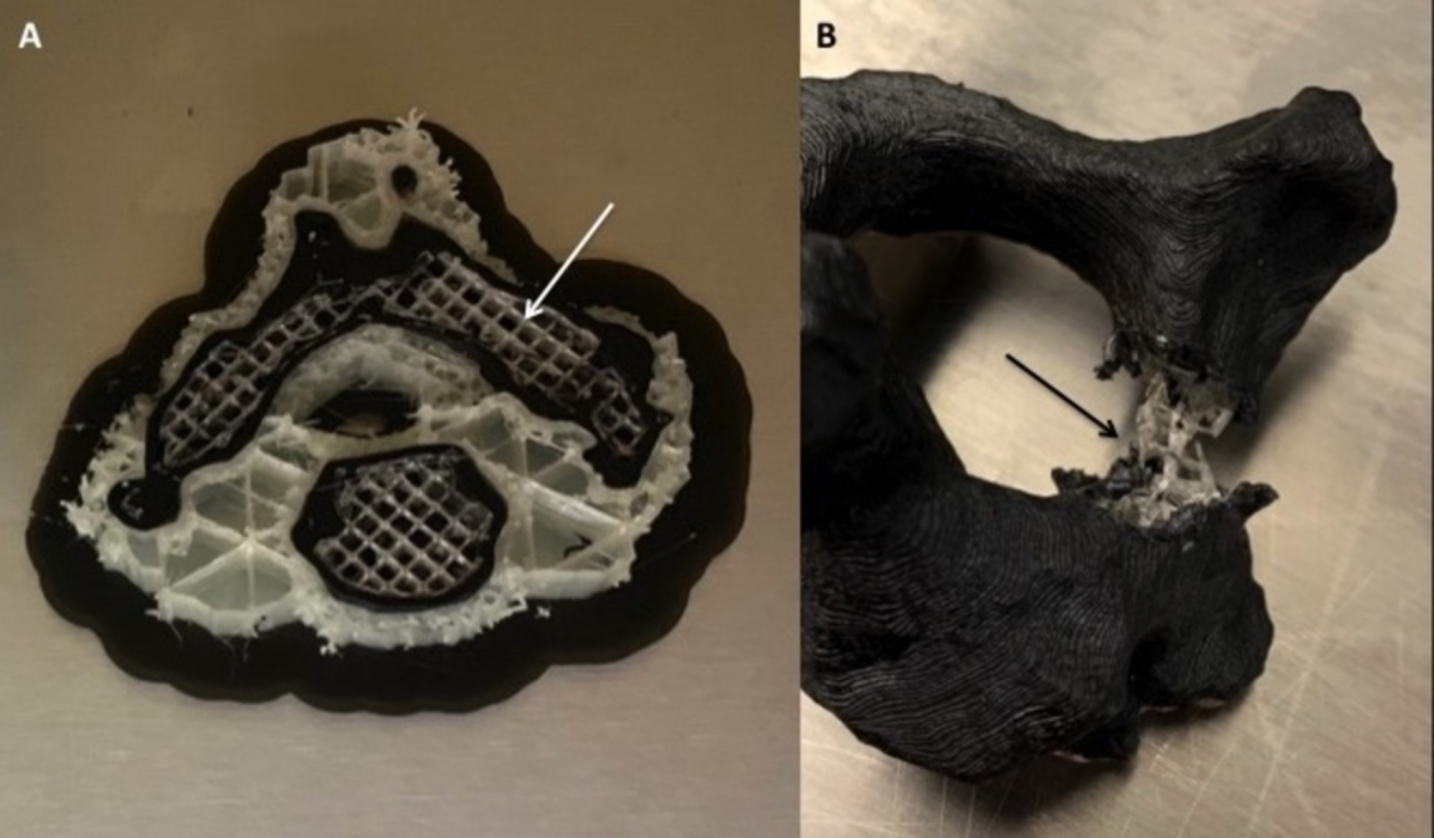
Production of the PLA/PVA model (A) The interface of the PLA (black) and PVA (white matrix, white arrow) is shown during a printing session. (B) The outer shell of “cortex” (PLA) is removed from the pedicle of a finished model demonstrating the inner matrix of PVA (black arrow). An advantage of dual-extrusion 3D printers is the ability to change material deposition within the same print. PLA, polylactic acid; PVA, polyvinyl alcohol

**Figure 3:**
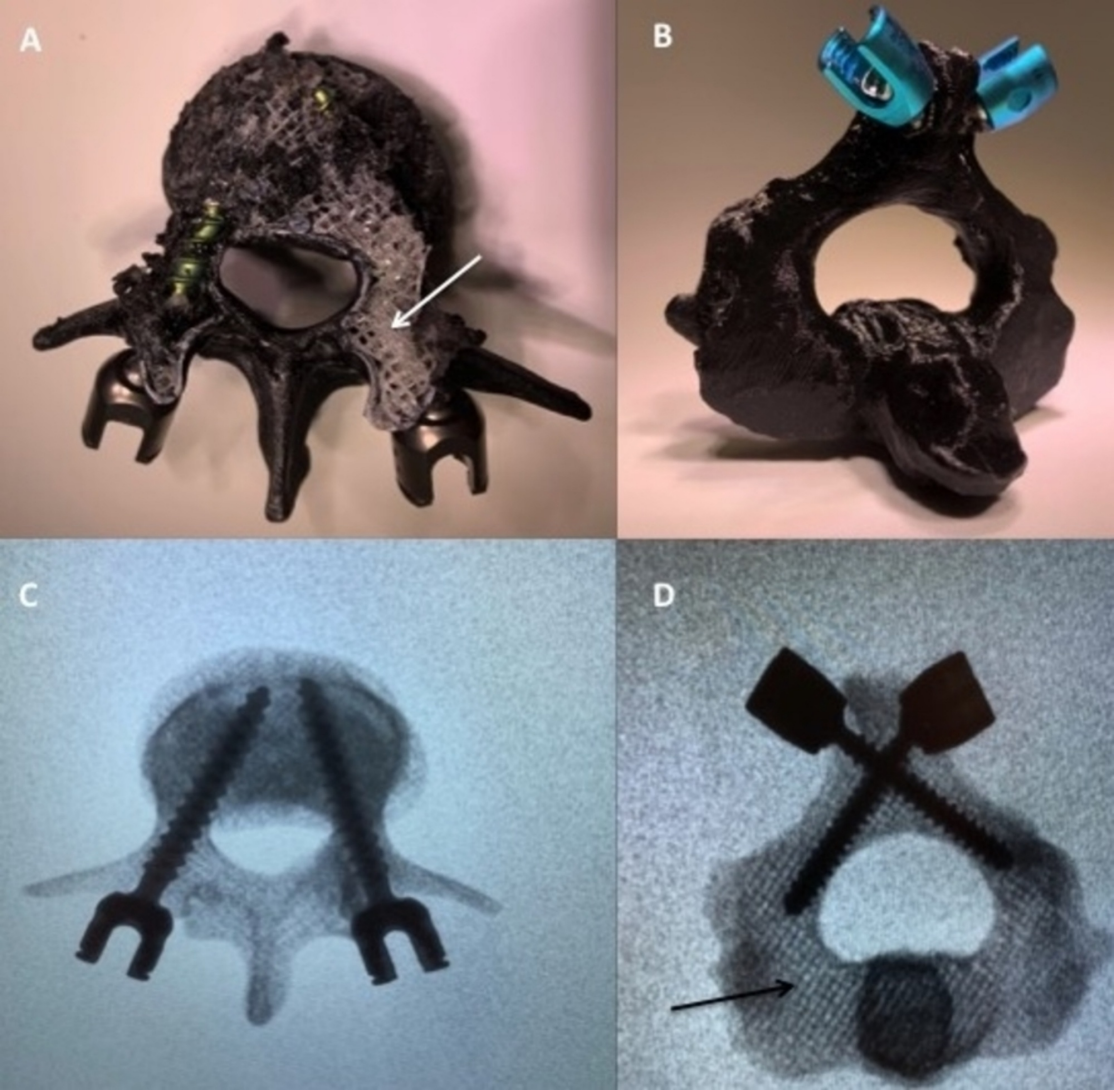
Instrumentation examples of experimental (PLA/PVA) models (A) L3 vertebrae with PLA shell removed demonstrating the spongy PVA matrix (white arrow). Pedicle screws are seen within the matrix. (B) Laminar screws placed in the C2 experimental model without model failure. (C & D) Fluoroscopic images obtained post instrumentation. The PVA infill matrix can be seen under X-ray (black arrow). PLA, polylactic acid; PVA, polyvinyl alcohol

Three lumbar vertebrae and twenty C2 vertebrae STL (stereolithograpy) file shells were edited using Meshmixer software then sliced using Cura software (Figure 1). A chosen 20% infill pattern with a small but uniform distribution on the software evenly dispersed the inlay PVA across the inner portion of the vertebral body, creating an inner matrix of spongy plastic PVA (Figure 2). A PLA outer shell was then programmed for the overlay, simulating cortical bone. The vertebrae were then horizontally printed on an Ultimaker S5 Dual Extrusion printer. Horizontal printing of these models perpendicular to the direction of planned probe and screw insertion into the pedicle allowed for maximal infill material contact by the instruments during pedicle access, thus increasing the tactile feedback and user experience. Instrumentation was placed in each of the experimental models using a freehand technique with a curved pedicle probe, tap, and screw in order to assess for tactile feedback and constitutional tolerance to instrumentation (Figure 3). Pedicle screws (6.5 x 45mm) were bilaterally placed in the lumbar vertebrae models, and both pedicle and laminar screws (4.5 x 30mm) were bilaterally placed in the C2 models. A control cohort of a polyurethane foam C2 and lumbar models, a single material (PLA) 3D printed lumbar and C2 model with the same inlay matrixing algorithm as the experimental specimens, and a resin lumbar model (printed on a Formlabs Form2 3D printer) were all instrumented using the same freehand technique and screw sizes for comparison.

## Results

**Figure 4:**
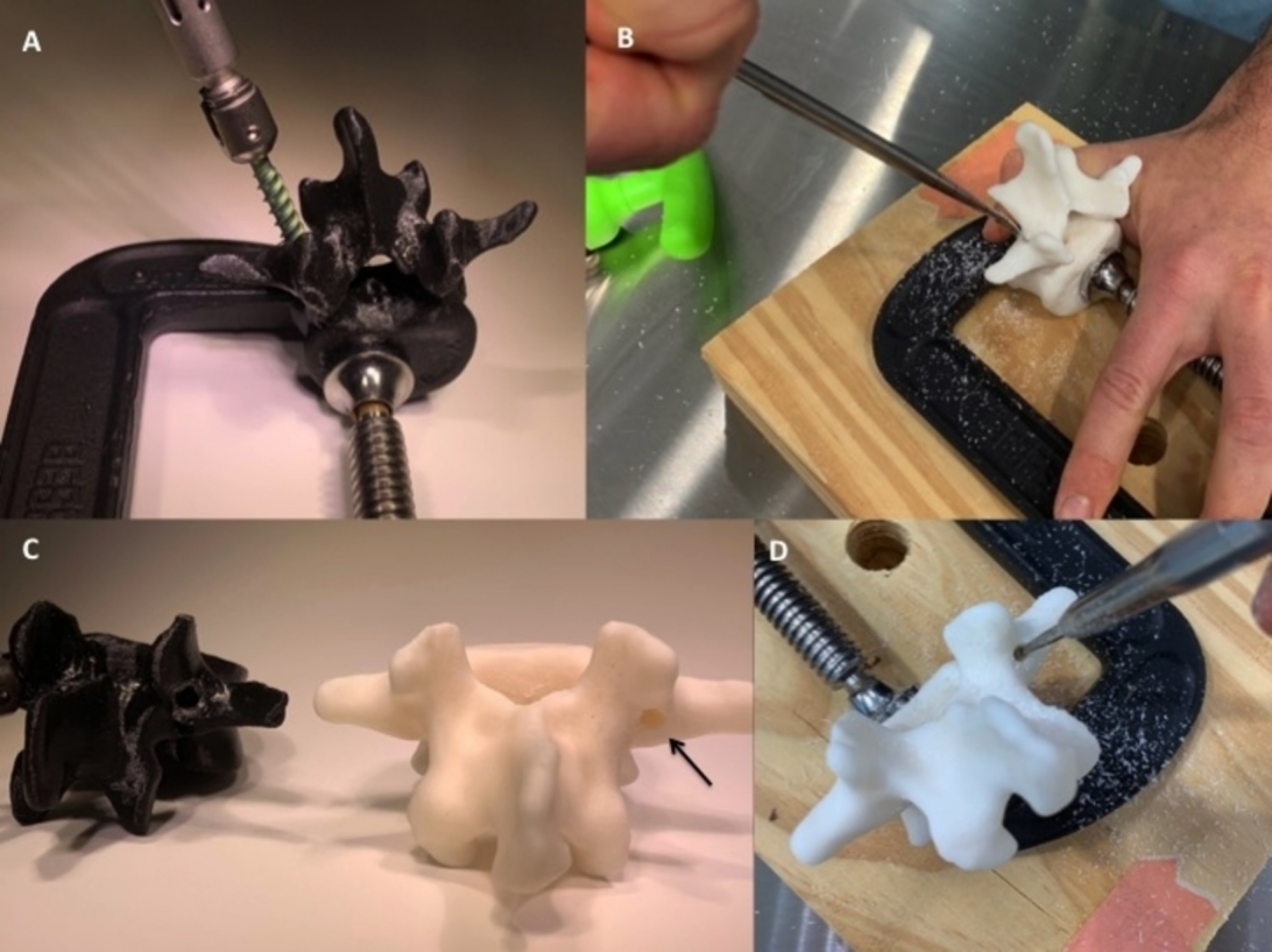
Instrumentation examples of the L3 PLA/PVA experimental model and a lumbar vertebrae resin print (A) The L3 PLA/PVA experimental model was successfully probed, tapped, and instrumented with a pedicle screw. (B) The resin print pedicle was unable to be accessed manually with the curved probe due to its solid properties. (C) The resin print (white) is shown to be completely solid (black arrow) when a pilot hole is drilled (D) with a high-speed burr. The pedicle of the resin model cracked after it was drilled and tapped. PLA, polylactic acid; PVA, polyvinyl alcohol

All of the PLA/PVA experimental models were successfully instrumented without hardware or material failures. There was an obvious need for increased manual force needed to properly cannulate the pedicle for the single material (PLA) print models compared with the experimental models. Anecdotally, the polyurethane foam model did not have any palpable cortico-cancellous difference. The resin print could not be manually probed and cracked during an attempt to cannulate the pedicle with a drill (Figure 4). Based on tactile feedback, the experimental dual material print (PLA/PVA) more accurately represented the sensation of in vivo instrumentation during pedicle probing, pedicle tapping, and screw placement. 

## Discussion

3D-printed materials that have been used to simulate vertebral bony anatomy for biomechanical testing and instrumentation include various resins, acryl butadiene styrene (ABS), nylon, polylactic acid (PLA), and thermoplastic urethane (TPU), with varying results [4,15-16]. Resin printing has become very popular in the medical community due to its ability to create finely detailed prints and vibrant colors. However, the material resins that have been used for previous spine models are very hard and must be drilled in some instances in order to instrument [15,17-18]. This technique does not reflect actual intraoperative procedure for spinal instrumentation and thus makes it suboptimal for simulation use. Another limitation of most previously published models is their constraint of a one material print. Literature suggests that decreasing the print infill percentage adequately simulates the density change of cortical bone to cancellous bone. These findings have been quantified with regards to subjective user feedback and biomechanical stress with good results [16,19-21]. A possible limitation of this modeling technique is the ultrastructure of the print does not completely simulate the complex characteristics of human vertebrae. Bone strength is composed of two properties: bone mass, or quantity of material, as well as the bone quality, or mechanical properties of the material. Previous spinal models have incorporated these parameters individually, but not collectively [4,20-21]. Dual extrusion FDM technology has wide applications for tissue engineering, scaffolding, and functional simulation. The full capabilities of this powerful technology have yet to be fully exploited in the medical community [22].

Our proposed dual material printing technique may be employed for the creation of any vertebral model. For the purposes of this feasibility study, our STL shells were limited to a lumbar and cervical vertebral model. These two shells were chosen due to their considerable anatomic differences and instrumentation placement methods in order to determine model viability when using this print method. Our results demonstrated that the concept of simultaneous dual material printing with PLA and PVA is a feasible method for replicating both the material and density difference in cortical and cancellous bone. There were no model failures in the experimental cohort, indicating resilience to instrumentation and capacity to be used as educational or biomechanical simulators.

## Conclusions

In order to create a spine model that better simulates the intraoperative in vivotactile feedback of the cortico-cancellous interface, we have created a novel vertebral simulator using simultaneous multiple material printing. This has large implications for the spinal instrumentation industry as well as resident training. This concept and design of simultaneous multi-material printing using our unique infill algorithm have not been yet reported in the medical literature. Further educational and biomechanical testing on our design is currently underway to establish this printing method as a new standard for spinal biomimetic modeling.
